# Supplementary material for: Identification of photoperiod-induced specific miRNAs in the adrenal glands of Sunite sheep (Ovis aries)
Source: Front Vet Sci. 2022 Jul 22;9:888207. doi: 10.3389/fvets.2022.888207 (PMC9354845; doi:10.3389/fvets.2022.888207)
Supplement: Supplementary file 1 [file Data_Sheet_1.ZIP › Supplementary file/miRDeep2 file.zip!/miRDeep2 file/Know_Structure/LP42A2/oar-mir-494.pdf]

miRBase precursor : oar-mir-494
Total read count : 11949
oar-miR-494-5p read count : 27
oar-miR-494-3p read count : 11918
remaining reads : 4

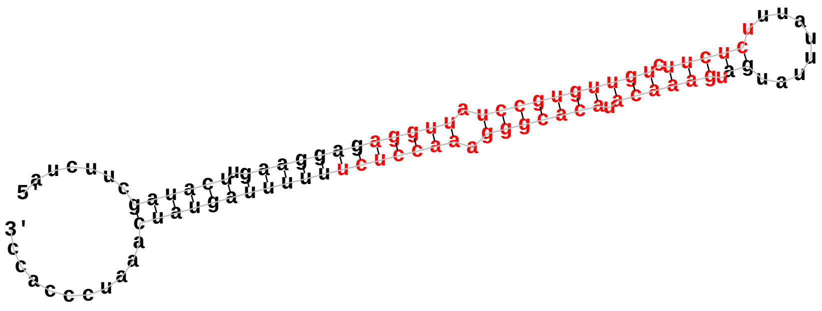

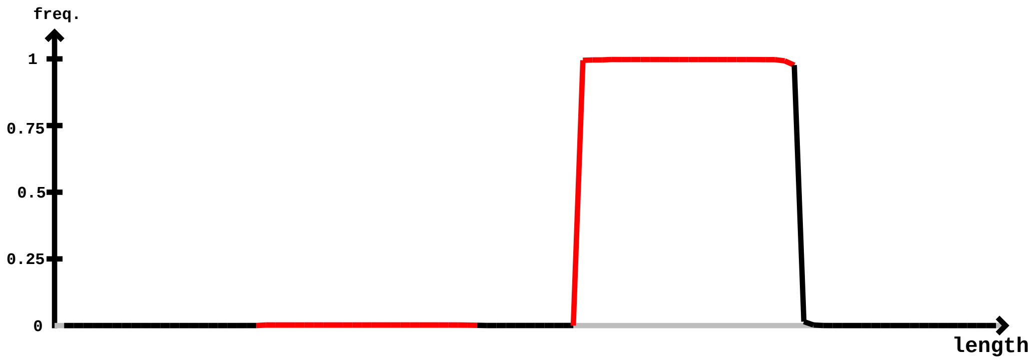


```
                                                  oar-miR-494-3p
                   oar-miR-494-5p
5'- aucuucgauacuugaaggagagguuauccguguugucuucucuuuauuuaugaugaaacauacacgggaaaccucuuuuuuaguaucaaaucccacc   -3'   exp
    ......((((((.((((((((((((.((((((((((.(((((.........)).)))))).))))))).)))))))))))))))))))..........   reads   mm   sample
    ..................agagguuauccguguugucuu..........................................................   1       0    seq
    ....................agguuauccguguugucuucu........................................................   6       0    seq
    ....................agguuauccguguugucuucuc.......................................................   10      0    seq
    ....................agguuauccguguugucuucucu......................................................   10      0    seq
    ...............................guugucuucucuuuauuuaugaugaa........................................   2       0    seq
    .............................................auuuaugaugaGacaua...................................   1       1    seq
    ................................................uaugaugaaacauacacgggaaaccucu.....................   1       0    seq
    .....................................................ugaaacauacacgggaaa..........................   1       0    seq
    .....................................................ugaaacauacacgggaaac.........................   4       0    seq
    .....................................................ugaaacauacacgggaaacc........................   1       0    seq
    .....................................................ugaaacauacacgggaaaccu.......................   53      0    seq
    .....................................................ugaaacauacacgggaaaccuc......................   191     0    seq
    .....................................................ugaaacauacacgggaaaccucu.....................   11474   0    seq
    .....................................................ugaaacauacacgggaaaccucuu....................   148     0    seq
    .....................................................ugaaacauacacgggaaaccucuuu...................   11      0    seq
    ......................................................gaaacauacacgggaaaccucu.....................   14      0    seq
    .......................................................aaacauacacgggaaaccucu.....................   3       0    seq
    ........................................................aacauacacgggaaaccucuu....................   2       0    seq
    ........................................................aacauacacgggaaaccucuuu...................   13      0    seq
    ........................................................aacauacacgggaaaccucuuuu..................   2       0    seq
    ..........................................................cauacacgggaaaccucu.....................   1       0    seq
```
